# Increased Urinary Concentration of C-Terminal Telopeptide of Type II Collagen and Pain by Radiographic Grade in Women with Knee Osteoarthritis in Northeastern Mexico: A Cross-Sectional Study

**DOI:** 10.1089/biores.2019.0003

**Published:** 2020-02-12

**Authors:** Francisco Javier García-Alvarado, Marisela del R. González-Martínez, Yolanda Jaramillo-Rodríguez, Héctor Alberto Delgado-Aguirre

**Affiliations:** ^1^ Facultad de Ciencias de la Salud, Universidad Juarez del Estado de Durango, Gomez Palacio, Mexico.; ^2^ Departamento de Microbiología, Facultad de Medicina, Universidad Autónoma de Coahuila, Torreón, México.; ^3^ Unidad Médica de Alta Especialidad No. 71, División de Investigación en Salud, Departamento de Patología General, Instituto Mexicano del Seguro Social, Torreón, México.; ^4^ Unidad Médica de Alta Especialidad No. 71, División de Investigación en Salud, Departamento de Trasplantes, Instituto Mexicano del Seguro Social, Torreón, México.

**Keywords:** biomarker, collagen type II, knee osteoarthritis, pain

## Abstract

Osteoarthritis (OA) of the knee causes disability, pain, and progressive destruction of cartilage in adult women. The objective of the study was to evaluate the concentrations of the urinary biomarker C-terminal telopeptide of type II collagen (CTX-II) and pain by radiographic grade in women with knee OA in northeastern Mexico: Cross-sectional study of 155 women with knee OA. Concentrations of biochemical parameters were evaluated and urine samples were collected to measure biomarker levels (uCTX-II) ng/mmol by competitive enzyme-linked immunoabsorbent assay (ELISA) technique and the Western Ontario and McMaster Universities Osteoarthritis Index (WOMAC) scale was used for pain classification; median age of 49 years and 29.1 kg/m^2^ of body mass index (BMI). uCTX-II biomarker levels were grade 2 (210.7 ng/mmol), grade 3 (314.8 ng/mmol), and grade 4 (478.8 ng/mmol) relative to Kellgren and Lawrence, uCTX-II levels were compared with WOMAC scale and presented significant statistical difference (*p* = 0.0001). An association of the biomarker CTX-II and an increase in BMI was found in female patients with knee OA (odds ratio = 1.01; 95% confidence interval 1.001–1.005; *p* = 0.047).This study demonstrates an increase in the levels of the biomarker uCTX-II, the degree of pain, and radiographic grade in women with knee OA in northeastern Mexico.

## Introduction

Osteoarthritis (OA) is the most prevalent joint disease causing appreciable disability in most adults >55 years of age.^1,2^ The knee is the most affected joint,^3^ manifested with pain, stiffness, and considerable functional disability,^4^ especially in women.^5,6^ Recent studies have revealed that, compared with healthy women, women with OA of the knee have decreased survival rates.^7^ The incidence increases with age.^8^ OA patients with increased body mass index (BMI) show an increased risk of comorbidities.^9,10^ The diagnosis of the disease is usually based on clinical symptoms and radiographic changes; X-ray images are the gold standard to confirm the clinical diagnosis and grade of disease.^11,12^ In contrast, the determination of biomarkers in OA is very useful to evaluate joint injury, one of the biomarkers most studied to determine the severity and degradation of cartilage in OA of the knee is the C-terminal telopeptide of type II collagen (CTX-II), which has been used as a marker of progression of cartilaginous injuries because it has a direct relationship with the radiological grade and clinical parameters of OA.^13^

Therefore, analysis of CTX-II levels appears to be an effective way to determine the collection of type II collagen and its relationship with the severity of disease.^14^ Recent studies have analyzed the correlation between the radiographic data of the knee OA and the clinical state of the affected joint by using both specific clinical scores and radiographic classification scales.^15^ Among the multiple pathophysiological mechanisms involved in OA, those related to the control of the sex hormone have attracted much attention, particularly those related to estrogens.^16^ The low level of estrogen production at menopause is associated with a relevant loss of muscle mass and, therefore, a significant deterioration in muscle performance and functional ability.^17^ The WOMAC (Western Ontario and McMaster Universities Osteoarthritis Index) is a scale that is valid for patients with OA of knee^18^ and analyzes three fundamental aspects such as pain, stiffness, and physical function.^19^ Higher WOMAC scores indicate greater severity of symptoms and functional limitations.

A discordance between symptoms and structure has been widely observed in OA, based on observations of weak correlations between radiographic severity and pain; therefore, the determination of some biomarkers may be very useful in clinical practice as a diagnostic procedure or prognosis of degenerative disease, consequently this study aims to evaluate the concentrations of urinary CTX-II biomarker, pain, and radiographic grade in Mestizo women with knee OA in northeastern Mexico.

## Materials and Methods

### Study population

A cross-sectional descriptive study was conducted of 155 women with OA knee Mestizas of Mexican nationality from the northeastern region of Mexico who had no parental relationship, who were selected in the department of orthopedics of the Clinic of the Mexican Institute of Social Security in the city of Torreon Coahuila, Mexico. The study was conducted in accordance with the Declaration of Helsinki and in compliance with the laws and regulations of the Mexican General Law of Health in Research for Health. The sample size was calculated based on the prevalence of Mestizo women with knee OA in northeastern Mexico. The test power calculation was considered the sample size of 155 patients with an alpha error of 5% with a test power of 80%.

### Radiographic evaluation of OA knee

All patients with knee OA met the American College of Rheumatology classification criteria for knee OA.^20^ Subsequently, structural damage of the knee was determined by anteroposterior and lateral radiographs in 30° flexion of the knee according to the radiological criteria of Kellgren and Lawrence,^21^ which were used to classify the radiographic grade of OA knee by a medical specialist in traumatology, which was categorized as mild (KL grade 2), moderate (KL grade 3), and severe (KL grade 4). Patients with inflammatory post-traumatic arthritis or any other rheumatic disease were excluded from the study.

### Sample collection

Women with confirmed diagnosis of knee OA had a morning urine sample (5–10 mL) collected and centrifuged at 2500 rpm/min for 20 min. The supernatant was transferred to a centrifuge tube and kept at −40°C for further analysis.

### Biochemical parameters

Concentrations of biochemical parameters such as cholesterol, triglycerides, uric acid, and glucose were evaluated by colorimetric techniques using a commercial kit for each analyte (Diagnostic Systems, Germany) and quantified by spectrophotometry (iMark, BioRad).

### Biomarker of CTX-II

The urine concentrations of the biomarker CTX-II ng/L were then measured in the Mestizo women in the study (*n* = 155). Using an enzyme-linked immunoabsorbent assay (competitive ELISA) (US Biological Life Science, Massachusetts) following the manufacturer's instructions. The CTX-II (ng/L) concentrations were standardized for total creatinine in urine (mmol/L), and the units for the adjusted uCTX-II concentration were left in ng/mmol.

### Knee pain evaluation

The WOMAC scale was used to classify patients' pain; knee pain was characterized by asking if patients who had knee pain and if it had occurred in >15 days of the month before the interview. Patients were classified as no knee pain (0), knee pain for <15 days per month (1), or knee pain for >15 days per month (2). Patients were informed of their X-ray radiographic classification ∼6 weeks after their interviews or images.

### Statistical analyses

A descriptive analysis of the studied patients was performed, data were summarized using frequencies and percentages for categorical variables as well as medians and interquartile range for continuous variables. The Kruskal–Wallis test was used to compare differences in urinary CTX-II concentrations, pain, and radiographic grade for continuous variables due to asymmetry of distributions. Logistic regression analysis was performed to evaluate BMI as a dependent variable and the independent variables such as the risk factors to knee OA of the study patients, the analyzed data were performed in the statistical program STATA 14 (Stata Corp., College Station, TX).

## Results

Included were 155 Mestizo women with OA knee according to the classification criteria of the American College of Rheumatology, with a median age of 49 years, with ranges from 44 to 62 years. The average BMI was 29.1 kg/m^2^ of the study population. The selected women presented an average concentration of 92.3 mg/dL glucose, 197.6 mg/dL cholesterol, 149.4 mg/dL triglycerides, and 6.3 mg/dL uric acid.

The WOMAC scale score was used to assess the symptomatology mainly to measure the pain perceived by patients with OA of the knee by means of a personal interview, which found that 23.8% of the patients had no knee pain, 50.3% had pain <15 days per month, and 25.8% had knee pain >15 days per month. The 155 individuals with knee OA were classified as (54.1%) with grade 2, (32.2%) grade 3 and (13.5%) grade 4 according to the Kellgren–Lawrence radiological scale (Table 1).

**Table 1.** Clinical Characteristics of Women with Knee Osteoarthritis in the Study

Characteristic	n (%)
Age (years)^a^	49 (44–62)
BMI (kg/m^2^)^a^	29.1 (29.4–31.2)
Uric acid (mg/dL)^a^	6.3
Glucose (mg/dL)^a^	92.3
Cholesterol (mg/dL)^a^	197.6
Triglycerides (mg/dL)^a^	149.4
Knee pain (*n*, %)^b^	
Without pain	37 (23.87)
Pain <15 days/month	78 (50.32)
Pain ≥15 days/month	40 (25.81)
Radiographic grade (K–L)^b^	
Grade 2	84 (54.19)
Grade 3	50 (32.26)
Grade 4	21 (13.55)

^a^ Median (IQR p25–p75).

^b^ No. of individuals (percentage).

BMI, body mass index; IQR, interquartile range.

**FIG. 1.**
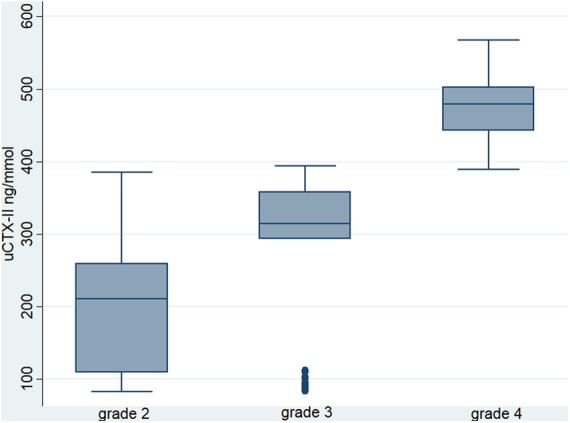
Relationship between radiological grade and concentrations of CTX-II in urine. CTX-II, C-terminal telopeptide of type II collagen.

The concentrations of the uCTX-II biomarker in urine were compared by radiographic grade. The group of OA knee patients had a median for grade 2 (210.7 ng/mmol), grade 3 (314.8 ng/mmol), and grade 4 (478.8 ng/mmol) relative to the Kellgren and Lawrence criteria. These results showed a statistical significance (*p* = 0.0001) with the radiological grade by Kruskal–Wallis analysis (Fig. 1, Table 2).

**Table 2.** Concentrations of the Biomarker C-Terminal Telopeptide of Type II Collagen in Women with Knee Osteoarthritis

Biomarker	Grade 2	Grade 3	Grade 4	p
	Median (IQR)^a^	Median (IQR)^a^	Median (IQR)^a^	
uCTX-II^b^	210.7 (108.9–259.3)	314.8 (293.4–358.1)	478.8 (442.5–502.6)	0.0001

^a^ Kruskal–Wallis test, median and IQR defined as the range from 25th percentile to 75th percentile.

^b^ uCTX-II urinary CTX-II concentrations adjusted for urine creatinine levels.

CTX-II, C-terminal telopeptide of type II collagen.

The urinary levels of uCTX-II were compared by degree of pain based on the metric scale of the WOMAC questionnaire. The group of patients without knee pain presented a median uCTX-II in urine of 105.8 ng/mmol, patients with knee pain for <15 days presented levels of 236.6 ng/mmol and knee pain for >15 days with urinary concentrations of 311.6 ng/mmol, showing statistical significance (*p* = 0.0001) by degree of pain (Table 3).

**Table 3.** Concentrations of the Biomarker C-Terminal Telopeptide of Type II Collagen by Degree of Pain of the Patients

Biomarker	Without pain	Pain <15 days	Pain >15 days	p
	Median (IQR)^a^	Median (IQR)^a^	Median (IQR)^a^	
uCTX-II	105.8 (99.9–246.5)	236.6 (111.6–358.1)	311.6 (237.5–384.9)	0.0001

^a^ Kruskal–Wallis test, median and IQR defined as the range from the 25th percentile to the 75th percentile.

The BMI variable was categorized with 54.8% overweight <29.9 kg/m^2^ and 45.2% with obesity BMI >30 kg/m^2^ as a dependent variable in women with knee OA, to perform a logistic regression model. Regarding urinary CTX-II, we found a strong association of the biomarker CTX-II and an increase in the BMI of women with knee OA (odds ratio = 1.01; 95% confidence interval 1.001–1.005; *p* = 0.047), but not in age, gender, pain, and biochemical parameters (Table 4).

**Table 4.** Logistic Regression of Obesity Risk for Women with Osteoarthritis Knee

Variable	Overweight (BMI <29.9 kg/m^2^), n = 85 (54.8%)	p		Obesity (BMI >30 Kg/m^2^), n = 70 (45.2%)	p
	OR crude (95% CI)			Adjusted OR (95% CI)	
Age	0.98 (0.95–1.01)	0.47		0.98 (0.94–1.02	0.29
Gender (female)	0.95 (0.50–1.80)	0.89		0.93 (0.49–1.76)	0.82
CTX-II	**1.01** (1.001–1.005)	**0.047**		**1.00** (1.0–1.007)	**0.046**
Glucose	1.00 (0.99–1.02)	0.46		1.03 (0.99–1.012)	0.44
Cholesterol	1.01 (0.98–1.04)	0.29		1.018 (0.98–1.05)	0.24
Triglycerides	1.03 (0.98–1.05)	0.47		1.014 (0.9–1.04)	0.36
Uric acid	0.70 (0.54–1.93)	0.76	0.68 (0.52–1.91)	0.71	
Pain	1		1		
Without pain					
>15 Days	1.84 (0.73–4.61)	0.19	1.77 (0.69–4.5)	0.22	
≤15 Days	1.66 (0.74–3.73)	0.21	1.59 (0.68–3.6)	0.27	

OR crude and OR adjusted for radiological grade. Bold values indicate statistical significance, *p* < 0.05.

CI, confidence interval; OR, odds ratio.

## Discussion

This study confirmed increased concentrations of the urinary CTX-II biomarker, degree of pain, and radiographic degree in mestizo women with knee OA in northeastern Mexico. In addition, the results showed a trend toward an increase in urinary uCTX-II levels based on the radiographic criteria of Kellgren and Lawrence. Recent studies describe different risk factors that are related to the onset and progression of OA of the knee such as aging, female gender, genetics, trauma, inflammation, and obesity.^5,22^ Srikanth et al. found that women are not only more likely to have OA than men, but also have more severe OA.^23^

A current increase in overweight and life expectancy is expected to increase the prevalence of OA.^24^ This poses OA as a serious public health problem for the future. In addition to age and weight, other risk factors for OA include female gender, genetics, poor nutrition, overuse of joints, trauma, muscle weakness, physical inactivity, and poor habitual movement patterns.^25,26^ Although the exact pathophysiology of OA has not yet been elucidated, it is now believed that altered joint load and cartilage metabolism are key factors in cartilage degradation and the subsequent development of pathology.^27^ The results of Framingham's study showed that women who had lost about 5 kg had a 50% reduction in the risk of developing symptomatic knee OA.

The same study also found that weight loss was strongly associated with a reduced risk of developing OA of radiographic knee.^28^ Our results found a strong association of the biomarker CTX-II and increased BMI in Mexican women with knee OA.

The knee pain score on the WOMAC scale is a widely validated tool and is applied considerably to assess patient-reported pain. In a population-based study, although the longitudinal trajectory of uCTX-II over time was positively associated with an index of knee stiffness, no significant association was found with the knee pain index and the WOMAC scale.^29^ Similarly, a study of patients with knee OA did not find a strong association between uCTX-II concentrations and WOMAC index scores.^30^ In contrast, Ishijima et al.^29^ reported higher concentrations of uCTX-II in people with early OA knee pain; similarly, our study showed an increase in uCTX-II concentrations and pain index in Mexican women with OA of the knee. Therefore, there is some evidence that uCTX-II concentrations may be elevated in people with knee symptoms, and more research is needed to explore the biological mechanisms that link uCTX-II concentrations with these clinical outcomes.

A recent study in middle-aged women without clinical knee disease showed higher levels of CTX-II associated with early structural changes in the knee.^31^ Also in another cross-sectional study involving women >60 years found that CTX-II levels were strongly correlated with the degree of OA knee.^32^ The mean uCTX-II concentration found among women with (KL grade 4) in this study was 478.8 ng/mmol, which is somewhat similar to the uCTX-II concentrations found in previous studies. Sowers et al.^27^ found a mean CTX-II concentration of 345 ng/mmol in 20 patients with severe knee OA (KL grade 3–4). Jung et al.,^31^ reported a mean concentration of 429 ng/mmol in 37 patients with knee OA. In addition, our results found a marked statistical difference between radiological grade and CTX-II concentrations in female patients with knee OA. In addition, our results found a marked statistical difference between radiological grade and CTX-II concentrations in female patients with knee OA. A previous study by Ding et al.^32^ showed that the severity of knee cartilage injury was significantly associated with CTX-II concentration. The urine sample taken from our study was done in the morning, you can see possible variations in urinary levels of CTX-II depending on the time and analysis of the sample, so the urinary levels of CTX-II were adjusted by urinary creatinine concentrations trying to control this variability. Owing to the limitations of the study, additional and improved multicenter studies with a larger number of patients are needed in the future that can confirm our findings to contribute to the research of patients with OA, the high complexity of the etiology and pathophysiology of the disease, as well as its high heterogeneity, suggest more research that will be useful to increase the confidence of these possible associations.

## Conclusions

This study demonstrated an increase in urine concentrations of the biomarker CTX-II, the degree of pain, and radiographic grade in mestizo women with knee OA in northeastern Mexico.

## Abbreviations Used

BMI: body mass index
CI: confidence interval
CTX-II: C-terminal telopeptide of type II collagen
OA: osteoarthritis
IQR: interquartile range
OR: odds ratio
WOMAC: Western Ontario and McMaster Universities Osteoarthritis Index
